# Biochemical Saliva Analysis and Caries Experience In Children With Black Tooth Stain

**DOI:** 10.3290/j.ohpd.c_2300

**Published:** 2025-11-06

**Authors:** Ayşe İpek Gün Topcu, Yıldırım Erdoğan, Şükrü Gökhan Elçi

**Affiliations:** a Ayşe İpek Gün Topcu Specialist in Paediatric Dentistry, Private Dental Practice, Denizli, Türkiye. Conceptualisation, methodology, data curation, formal analysis, visualisation, writing – original draft, review and editing; b Yildirim Erdoğan Associate Professor, Department of Paediatric Dentistry, Faculty of Dentistry, Pamukkale University, Denizli, Türkiye. Conceptualisation, methodology, data curation, formal analysis, visualisation, supervision, project administration, resources, writing – original draft, review and editing.; c Şükrü Gökhan Elçi Associate Professor, Department of Chemistry, Faculty of Science, Dokuz Eylül University, İzmir, Türkiye. Writing – review and editing.

**Keywords:** black tooth stain, dental caries, mixed dentition, salivary biochemistry, trace elements

## Abstract

**Purpose:**

Black tooth stain (BTS) is an extrinsic discolouration that typically appears along the cervical third of the buccal and/or lingual surfaces of both primary and permanent teeth, predominantly in prepubertal individuals. This study aimed to investigate the biochemical properties of saliva and assess caries experience in children with and without BTS during the mixed–dentition period.

**Materials and Methods:**

A total of 120 children aged 7–12 years were enrolled and divided equally into BTS(+) and BTS(–) groups. Comprehensive dental examinations were conducted using dft/DMFT indices and International Caries Detection and Assessment System (ICDAS) II criteria to evaluate caries status and the presence of BTS. Unstimulated whole saliva samples were collected and analysed for flow rate, pH, buffering capacity, and ion concentrations (Cu, Fe, P, Ca, Mg, Zn, and Mn) using an inductively coupled plasma mass spectrometry (ICP-MS) method. The obtained data were analysed and compared between the groups.

**Results:**

The BTS(+) group demonstrated significantly lower dft scores and fewer decayed primary teeth compared to the BTS(–) group (P <0.05). A higher proportion of caries-free teeth was also observed in the BTS(+) group. Additionally, a statistically significant difference was found between the groups in terms of ICDAS II caries classification (P = 0.048). Salivary analysis showed significantly elevated manganese (Mn) and zinc (Zn) levels in the BTS(+) group, while no significant differences were found in pH, buffering capacity, flow rate, or other salivary ions. No significant correlations were observed between the severity of BTS and age, salivary parameters, or caries indices.

**Conclusion:**

Children with black tooth stain exhibited a lower caries experience, which may be associated with the anticariogenic properties of higher salivary Mn and Zn levels. Additionally, elevated Mn concentrations might play a role in the formation of BTS.

Tooth discolouration is a common dental finding associated with clinical and aesthetic problems. According to its location, it is divided into three groups: intrinsic, extrinsic, and internalised colourations. Intrinsic discolourations occur when pigmented material penetrates the tooth structure. Extrinsic discolourations are attachments on the tooth surface or acquired pellicle. Internalised stains occur when external stains penetrate the dentin through tooth flaws, such as surface cracks.^12,32,37^

Black tooth stain (BTS) is a distinctive extrinsic dental discolouration that typically appears along the third cervical line of the buccal and/or lingual surfaces of both primary and permanent teeth, characterised by discontinuous black dots or dark lines deposited on the crown that, in severe cases, may cover most of the tooth surface or even affect the pits and grooves.^5,30,40^ Studies report that BTS affects between 2.4% and 26% of the population, with a similar distribution observed between both genders.^5,17,29^ This particular type of pigmentation has been considered a special form of dental plaque, because it contains insoluble iron salt and high amounts of calcium and phosphate.^31^ Analysis of the black material indicates that it is a ferric salt, probably ferric sulfide, formed by the interaction of bacterial hydrogen sulfide with iron in saliva or gingival crevicular fluid.^27^ Supporting these classical approaches, recent studies have demonstrated that BTS samples contain significantly higher concentrations of iron than normal plaque,^38^ and that BTS plaques are enriched with genes and protein components that play a crucial role in bacterial iron uptake.^39^ Although the fundamental microbial characteristics and relationships between microbial communities and BTS remain incompletely understood, its formation appears to be related to a core microbiome that includes *Actinomyces* (especially *A. naeslundii*), *Prevotella nigrescens*, *Pseudopropionibacterium*, *Leptotrichia*, *Neisseria*, and *Rothia*.^5^

BTS is a common finding in children; however, it can also be seen in adults.^27^ Due to the poor aesthetic appearance and the ineffectiveness of conventional oral hygiene methods, such as using a toothbrush and toothpaste, black tooth stains cause anxiety in parents and can have significant effects on a child’s self-confidence.^12^ However, even after professional cleaning, black stains frequently reappear within months, likely due to the failure to inhibit the underlying mechanism.^25^

The presence of black tooth stain in children has been commonly associated with a low-caries experience.^7,10,13,15,35^ In some studies of children with black tooth stain, saliva has higher calcium-phosphate concentrations and higher saliva buffering capacity and it was claimed that the parameters that prevent caries formation and presence of non-cariogenic plaque may be associated with low-caries experience in these children.^8,34^ Although there are many prevalence and microbiological analysis studies on black tooth stain, biochemical analysis studies with saliva samples are limited.^8,21,23,34^

During the mixed–dentition stage, newly erupting permanent teeth have immature enamel, increasing caries risk.^3^ At this stage, saliva’s protective functions, such as acid neutralisation and enamel remineralisation, become especially crucial, making salivary analysis important for identifying factors influencing caries susceptibility during this transitional period.^18^ The aim of this study is to biochemically examine the saliva samples of children in the mixed dentition with and without BTS, and to determine the relationship between BTS, salivary parameters, and caries experiences.

## MATERIALS AND METHODS

This study was approved by the Ethics Committee of Pamukkale University (date: 25 May 2021, no: E-60116787-020-56584) and conducted in accordance with ethical principles of the Declaration of Helsinki. Before the study was initiated, all parents or guardians were informed of the objective of the investigation, and written consent was obtained for the participation of each child in this study.

Power analysis using the G*power 3.1 program indicated that to achieve 80% power with 95% confidence, a minimum of 36 individuals should be included in each group.8 Therefore, a minimum of 72 children, divided into two groups based on black tooth stains, needed to participate in the research.

### Subject Selection

The study involved 120 children, aged 7–12, in the mixed dentition era with no health issues, who visited the Pamukkale University Faculty of Dentistry, Department of Pediatric Dentistry Clinic, between September and November 2021.

The study excluded children who had systemic, genetic, or syndromic diseases, those who had taken antibiotics in the last 15 days, those who had used substances such as iron or chlorhexidine that could cause external discolouration, and those diagnosed with periodontal disease based on clinical findings such as gingival inflammation, periodontal pocket formation, or clinical attachment loss. The children were placed into two equal groups: one group with a black tooth stain, labelled as BTS(+), and another group without a stain, labelled as BTS(–).

### Clinical Examination

Dental examinations were performed by a calibrated paediatric dentist (AIGT) to evaluate black stains and dental caries, in the dental unit under reflector light using an intra-oral mouth mirror and a ball-end periodontal probe (WHO probe), leaving an inspection distance of 25 cm between the occlusal surface of the teeth and the eye in accordance with World Health Organization (WHO) guidelines.^22^ The teeth were assessed by subjecting them to a drying process followed by a 5-s application of compressed air.

**Fig 1:**
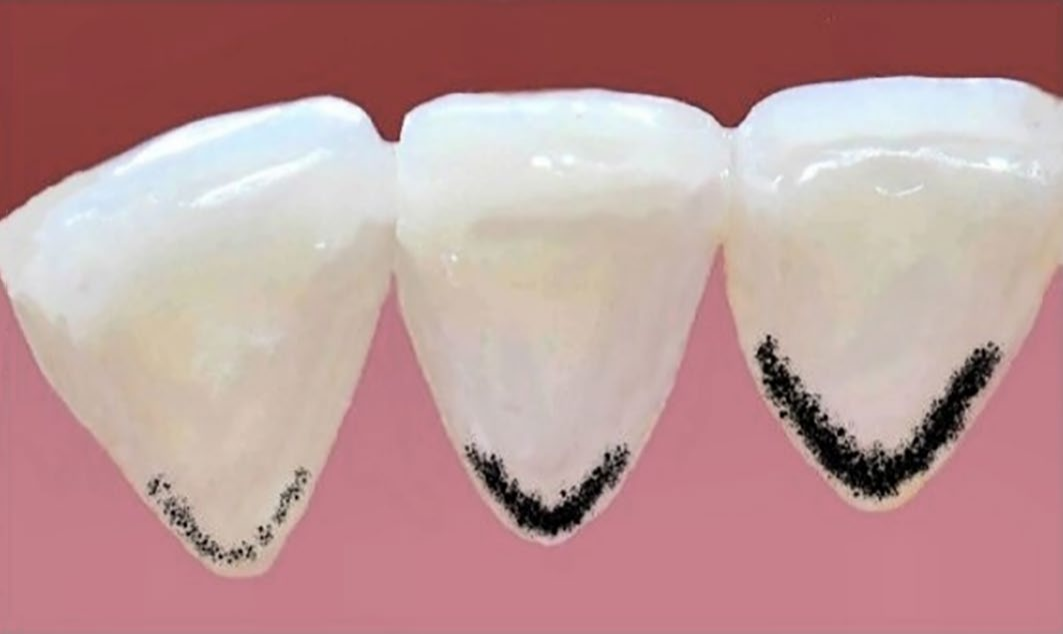
Representative images of black stain classification based on the criteria proposed by Gasparetto et al.^4^ The teeth are shown from left to right corresponding to Score 1 (mild), Score 2 (moderate), and Score 3 (severe).

Caries-related records were taken using the dft/DMFT (decayed and filled primary teeth/decayed, missing, and filled permanent teeth) and ICDAS II (International Caries Detection and Assessment System II) indices. ICDAS II Code (0) has indicated ‘non-carious’; ICDAS II Code (1, 2) were reversible lesions (white spot lesions), and ICDAS II Code (3, 4, 5, 6) were saved as ‘cavitated carious’ teeth.^2,24^ The clinical diagnosis of the black stains was performed according to the criteria of Gasparetto et al^10^ (Table 1, Fig 1). Score 1 corresponded to the presence of pigmented dots or thin lines with incomplete coalescence parallel to the gingival margin; score 2 corresponded to continuous pigmented lines, which were easily observed and limited to half of the cervical third of the tooth surface; score 3 corresponded to the presence of pigmented stains extending beyond half of the cervical third of the tooth surface.

**Table 1:** Classification criteria for black tooth stain according to Gasparetto et al^10^ (2003)

Score	Criteria
**Score 1**	Incomplete line or discontinuous pigmented dots parallel to the gingival margin. Stain involves less than one-third of the cervical third of the tooth surfaces.
**Score 2**	Continuous pigmented line limited to the cervical third of the tooth crown.
**Score 3**	Pigmented line extends beyond the cervical third, covering the middle third or more of the crown surface.


### Saliva Sampling Procedure and Biochemical Saliva Analysis

Unstimulated whole saliva samples of the patients were collected 2 h after a standard breakfast between 9:00 and 12:00 in the morning to avoid being affected by circadian rhythm changes. Patients were placed in an upright and comfortable position on the chair in the unit. Patients were instructed to abstain from swallowing for a duration of 10 min, refrain from making any movements with their lips or tongue, and let the saliva that had gathered in their mouth be drained passively into the plastic tubes they were holding.

**Fig 2:**
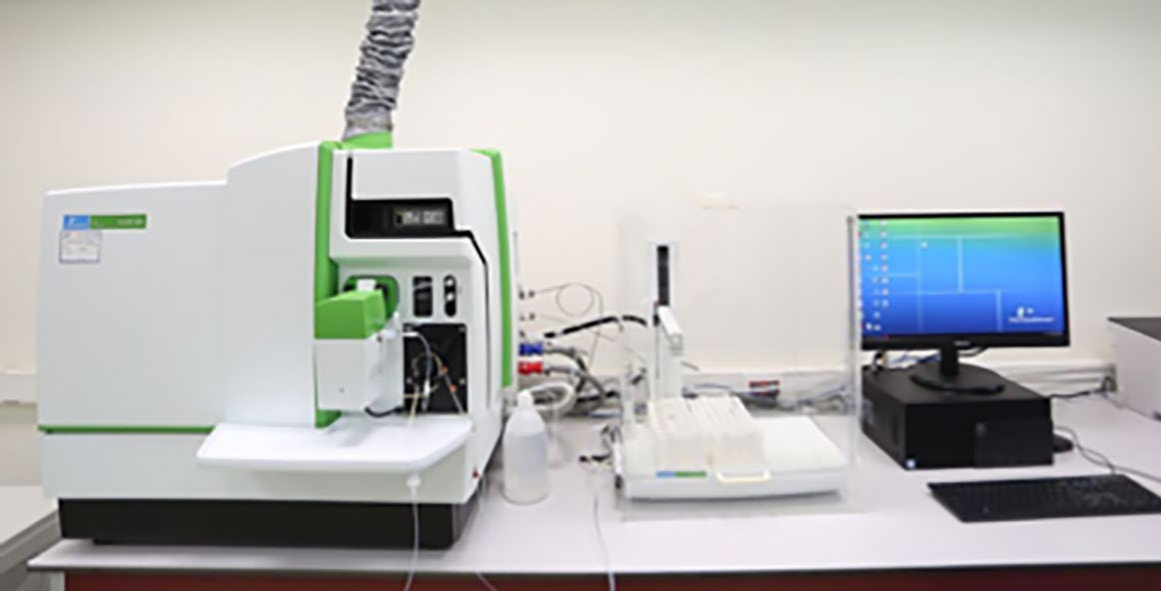
Inductively coupled plasma mass spectrometry (ICP-MS) device used for elemental analysis (PerkinElmer NexION® 2000, Waltham, MA, USA).

The saliva samples were partitioned into two distinct tubes for the purpose of measuring saliva parameters and conducting ion analysis. The saliva flow rate, pH, and saliva buffering capacity values were measured within 30 min of sample collection. The remaining samples were maintained at +4°C in a medical refrigerator (Frenox CGL2-M, İstanbul, Turkey) until the day of saliva ion analysis. The average salivary flow rate was determined by dividing the total volume by the time required to collect the sample. The saliva samples were analysed using a personal pH meter (Thermo Scientific ™ Orion Star ™ A111, Chelmsford, MA, USA) during a 30-min timeframe. The Ericsson method^6^ was used to determine the buffering capacity. The amount of Cu, Fe, P, Ca, Mg, Zn, and Mn ions in saliva samples was calculated using an ICP-MS equipment (PerkinElmer’s NexION® 2000, Waltham, MA, USA) (Fig 2).

### Statistical Analysis

The collected data were analysed using SPSS 21.0 software (SPSS, Chicago, IL, USA). For comparing groups consisting of two groups, the independent two-sample t-test was employed when the quantitative variables met the assumption of normal distribution. In cases where the normal distribution assumption was not met, the Mann–Whitney U test was used. For comparisons involving more than two groups, the Kruskal–Wallis analysis of variance was utilised. The Dunn’s test was employed as a multiple comparison test. Spearman correlation analysis was utilised to ascertain the associations between quantitative variables. Chi-square analysis was used to examine the relationships between category variables. The significance level was established at P <0.05.

## RESULTS

A cohort comprising 120 children (58 females, 62 males) was analysed for this study. The mean age of the participants was 9.53 ± 1.66 years (minimum age: 7 years, maximum age: 12 years). Among the 60 children with BTS, 31 (51.67%) were female and 29 (48.33%) were male, whereas within the control group of 60 children without BTS, 27 (45%) were female and 33 (55%) were male. Statistical analysis revealed no significant correlation between the presence of BTS and gender (P = 0.465).

According to the results obtained from our analyses, the dft (decayed, missing, filled teeth) index scores are significantly lower in BTS(+) children compared to BTS(–) children (P <0.05). The number of decayed teeth (dt) in the primary dentition is significantly lower in the BTS(+) group than in the BTS(–) group (P <0.05). However, the difference in DMFT indices between the groups is not statistically significant (P = 0.594) (Table 2).

**Table 2:** Caries distribution between BS(+) and BS(–) groups according to dft/DMFT indices

Variable	BS(+) (n = 60)	BS(–) (n = 60)	+ P value
**Dt**	0.0 (0.0–4.0)	1.0 (0.0–8.0)	0.028
**Ft**	1.0 (0.0–7.0)	1.0 (0.0–7.0)	0.664
**Dft**	2.0 (0.0–7.0)	3.0 (0.0–9.0)	0.046
**DT**	0.0 (0.0–3.0)	1.0 (0.0–4.0)	0.133
**MT**	0.0 (0.0–2.0)	0.0 (0.0–2.0)	0.180
**FT**	0.0 (0.0–4.0)	0.0 (0.0–7.0)	0.933
**DMFT**	1.0 (0.0–5.0)	1.0 (0.0–9.0)	0.594
+ Data are presented as median (min–max). Statistical comparisons were performed using the Mann–Whitney U test.

The ICDAS II Code (3, 4, 5, 6) scores are considerably higher than the Code (0) and Code (1, 2) scores in both the BTS(+) and BTS(–) groups (P <0.05). The BTS(+) group has a higher number of ICDAS II Code (0) scores, which indicate no caries, compared to the BTS(–) group (Table 3).

**Table 3:** Distribution and percentages of ICDAS II codes between BS(+) and BS(–) groups

Variable ICDAS II (Code)	Groups	+ P value
BS(+) N (%)	BS(–) N (%)
(0)	4 (6.7%)	1 (1.7%)	**0.048**
(1, 2)	21 (35.0%)	12 (20%)
(3, 4, 5, 6)	35 (58.3%)	47 (78.3%)
+ Pearson’s chi-square test.

Salivary Mn and Zn ions in BTS(+) children are found to be significantly higher than in BTS(–) children. However, there are no statistically significant differences in salivary pH, saliva buffering capacity (SBC), saliva flow rate (SFR), Mg, P, Ca, Fe, and Cu variables between BTS(+) and BTS(–) groups (P >0.05) (Table 4).

**Table 4:** Comparison of salivary parameters between BS(+) and BS(–) groups

Variable	Groups	+ P value
BS(+) (n = 60)	BS(–) (n = 60)
**pH**	6.86 ± 0.26	6.90 ± 0.30	0.392
**SBC**	4.17 ± 1.02	4.15 ± 1.01	0.911
**SFR**	0.21 (0.01–1.66)	0.26 (0.03–1.38)	0.621
**Mg**	4.38 (1.10–19.12)	4.57 (0.95–10.8)	0.616
**P**	188.83 (52.58–439.75)	193.68 (29.65–478.10)	0.946
**Ca**	6.61 (2.24–23.17)	7.27 (2.21–19.87)	0.183
**Mn**	0.02 (0.00–0.59)	0.01 (0.00–0.11)	0.002
**Fe**	1.36 (0.22–8.01)	1.41 (0.27–20.30)	0.694
**Cu**	0.12 (0.00–2.11)	0.09 (0.01–9.97)	0.078
**Zn**	0.75 (0.02–4.27)	0.57 (0.01–6.20)	0.021
+ Data are expressed as mean ± standard deviation or median (min–max), as appropriate.Variables presented as means were analysed using Student’s t-test, while those presented as medians were analysed using the Mann–Whitney U test.

There is no statistically significant difference between the severity of black stain and age, salivary parameters, salivary ions, and caries indices (P >0.05) (Table 5).

**Table 5:** Relationship between severity of black stain and age, caries indices, and saliva parameters

Variable	Severity of black stain	+ P value
Score 1 (n = 12)	Score 2 (n = 21)	Score 3 (n = 27)
**Age**	9.50 (8.0–12.0)	10.0 (7.0–12.0)	10.0 (7.0–12.0)	0.633
**pH**	6.79 (6.39–7.27)	6.88 (6.29–7.42)	6.87 (6.38–7.43)	0.821
**SBC**	3.82 (2.97–5.18)	4.70 (2.65–6.31)	3.68 (2.84–6.87)	0.233
**SFR**	0.28 (0.15–0.83)	0.30 (0.01–1.66)	0.18 (0.09–1.66)	0.54
**dft**	2.5 (0.0–7.0)	1.0 (0.0–7.0)	2.0 (0.0–6.0)	0.909
**DMFT**	1.0 (0.0–4.0)	2.0 (0.0–5.0)	1.0 (0.0–5.0)	0.096
**Mg**	3.5 (1.1–8.05)	5.11 (2.3–17.2)	4.4 (1.8–19.1)	0.327
**P**	152.2 (78.2–297.4)	195.51 (84.88–439.8)	186.1 (52.58–334.01)	0.194
**Ca**	5.4 (2.7–7.7)	7.4 (2.2–23.2)	7.0 (2.5–17.8)	0.062
**Mn**	0.01 (0.0–0.06)	0.02 (0.0–0.14)	0.02 (0.0–0.59)	0.166
**Fe**	1.44 (0.28–4.05)	1.36 (0.37–7.57)	1.33 (0.22–8.01)	0.524
**Cu**	0.08 (0.06–0.84)	0.16 (0.0–1.39)	0.12 (0.01–2.11)	0.332
**Zn**	0.69 (0.39–2.09)	0.92 (0.02–2.38)	0.68 (0.07–4.27)	0.439
+ Data are expressed as median (min–max). Statistical comparisons among groups were performed using the Kruskal–Wallis test.

## DISCUSSION

The presence of black tooth stains in children can significantly impact their social interactions and psychological well-being, potentially leading to a loss of self-confidence. Over recent years, heightened parental awareness regarding oral and dental health, coupled with increasing aesthetic expectations, has led to a surge in interest in the diagnosis and treatment of tooth discolouration.^36^ Although black tooth stains are often perceived solely as aesthetic issues, research suggests that their presence is associated with a reduced prevalence of dental caries, implying broader implications for oral health.^7,10,13,15,35^ It has been claimed that there is a potential association between black dental discolouration and caries prevalence, and that this relationship is mediated by alterations in oral microflora as well as variations in saliva composition.^26^

The inverse association between black tooth stain (BTS) and dental caries experience has been consistently demonstrated across all age groups.^4,19^ This relationship may be explained by the fact that plaque associated with black stain is typically dominated by less cariogenic bacteria, such as *Actinomyces* species, suggesting that black stain might reflect a healthier oral microbiological environment.^31,40^ Additionally, it has been proposed that higher salivary pH^40^, greater buffering capacity,^8^ or increased iron content^4^ may contribute to a less cariogenic oral environment. Nevertheless, some authors have emphasised caution, noting that current evidence primarily derives from cross-sectional studies, which prevents drawing definitive conclusions regarding causality. They advocate for longitudinal and interventional studies to clarify whether black stain actively contributes to caries prevention or merely represents a coincidental indicator of inherently low caries risk.^24^ It has been suggested that children who naturally exhibit lower caries risk due to genetic factors, diet, oral hygiene habits, fluoride exposure, or microbiota composition may simply be more likely to develop black-stained plaque. Furthermore, particularly in younger age groups, although a numerically inverse trend between black stain and caries experience has been observed in most studies, a few studies have reported no statistically significant association,^7,9^ and only one study has indicated a potential positive correlation.^20^

In the present study, conducted among children with black tooth stain, the combined application of the dft/DMFT indices, which quantify caries experience based on clinically detectable cavitated lesions, and the ICDAS II index, which enables the identification of early-stage, non-cavitated enamel lesions, allows for a more detailed and comprehensive assessment of the caries continuum.

Epidemiological studies examining the relationship between BTS and dental caries have predominantly employed traditional caries assessment indices, such as the WHO criteria.^19,40^ The ICDAS II system, known for its greater sensitivity and detailed scoring, has been used in only two prior studies addressing this relationship. Pehlivan et al^24^ in their study involving teeth with and without black extrinsic stains from 10 adults, reported that teeth exhibiting black stain had a significantly higher proportion of sound surfaces (ICDAS Code 0) and overall lower caries levels compared to non-stained teeth, indicating that black stain presence is associated with reduced caries experience. In contrast to most studies utilising traditional methods, Muthu et al^20^ found that children aged 0–3 years with extrinsic tooth stains exhibited 1.13 times higher caries prevalence in primary teeth compared to their non-stained counterparts, with statistically significant differences observed for both fissure and smooth surfaces.

Our study specifically focused on mixed dentition, a distinct developmental stage compared to the primary and permanent dentitions evaluated in the aforementioned studies using ICDAS II criteria. According to our findings, there was a statistically significant difference between BTS(+) and BTS(–) groups in terms of ICDAS II caries classification (P = 0.048). Teeth categorised as sound (ICDAS code 0) or presenting early-stage lesions (ICDAS codes 1 and 2) were more prevalent in the BTS(+) group, while advanced-stage lesions (ICDAS codes 3–6) occurred more frequently in the BTS(–) group. These results suggest that black tooth stain may be associated with lower caries severity. The discrepancies observed across studies may be attributable to variations in the age groups selected, differences in individual susceptibility factors, and methodological differences in applying the diagnostic criteria.

Our finding of significantly lower dft scores in BTS(+) children, coupled with no DMFT difference, mirrors the pattern reported by Garan et al.^8^ This suggests that black stain’s caries-suppressive association is most pronounced in primary dentition. Prior studies support this trend: Heinrich-Weltzien et al^13^ observed that even in predominantly permanent dentitions (12-year-olds), children with black stain had markedly lower caries experience than those without, and Gasparetto et al^10^ similarly found an inverse correlation between black stains and DMFT in schoolchildren. Notably, our cohort did not show a significant DMFT difference, aligning with Garan’s findings and hinting that the protective effect of BTS association between BTS and reduced caries might diminish as children transition to permanent teeth. The stronger caries resistance in primary teeth could stem from physiological factors, including a black stain-associated plaque with a microbial profile less conducive to caries, such as very low *Streptococcus mutans* levels^14^ resulting from competitive flora, and enhanced salivary defences in BTS(+) children, notably higher buffering capacity and elevated calcium levels.^8^ These factors would disproportionately benefit the These factors could disproportionately influence the caries experience of the more vulnerable enamel of primary teeth. Additionally, behavioural patterns might play a role; children with BTS often have good oral hygiene or lower sugar exposure, which may confer early protection against caries, which may be associated with lower caries experience that tapers off in later years as diet and habits change.

Despite the clear caries disparity, our salivary biochemical analyses revealed only subtle differences between BTS(+) and BTS(–) children. No significant group differences were detected in salivary pH, buffering capacity, flow rate, or major mineral ion levels (calcium and phosphate). This outcome stands in contrast to several earlier studies that documented a distinct salivary profile associated with BTS. Surdacka’s pioneering investigations in the 1980s found that children with ‘black tartar’ had a mineral-rich, alkaline saliva: significantly higher levels of total calcium and inorganic phosphate, along with elevated sodium, copper, and total protein, compared with controls.^33,34^ Stimulated saliva pH in the BTS group was also higher than in non-BTS children, whereas flow rates were comparable. Surdacka et al^34^ interpreted these findings to mean that BTS-prone children exhibit salivary characteristics typical of low-caries individuals, such as abundant mineral content and enhanced buffering capacity. Our study did not replicate the calcium/phosphate elevations or pH rise reported by Surdacka. This discrepancy may be due to differences in saliva collection (unstimulated in our study vs stimulated in Surdacka’s), age range (7–12 in our study vs 4–16 in Surdacka’s), or geographic factors affecting diet and trace element exposure. It is noteworthy, however, that we observed a trend towards higher salivary copper in the BTS(+) group, though not statistically significant, in agreement with Surdacka’s finding of elevated Cu in black stain. This study suggests copper and other trace elements might contribute to black stain formation. Thus, while our results differ in magnitude from Surdacka’s, they do not entirely refute the notion that BTS is associated with an altered salivary mineral milieu.

Our findings are more closely aligned with those of Garan et al^8^ (2012) in certain aspects. Garan and colleagues compared salivary factors in Turkish children with and without black stain, and reported significantly higher buffering capacity and calcium in the BTS group, with no difference in phosphate or resting pH. They also noted a paradoxically lower unstimulated flow rate in BTS (+) children, a result that we did not observe; in our data, flow rates were similar between groups. Like our study, Garan et al found no significant pH difference – mean salivary pH was alkaline in both BTS(+) and BTS(–) children (~7.7). Importantly, this study confirmed that the mean dft was markedly lower in the black stain group, mirroring our caries findings. They concluded that the low caries propensity in BTS children may be associated with their elevated salivary calcium and superior buffering capacity. Our results only partially support this hypothesis, although we did not detect a significant Ca or buffer advantage in the BTS cohort; the BTS(+) children did show numerically higher mean calcium and buffering values than controls. The direction of these differences is consistent with Garan’s report and with the broader pattern in the literature,^28^ suggesting that a larger sample or controlled conditions might reveal small but meaningful elevations. One notable divergence was salivary flow: Garan^8^ observed reduced flow in BTS children, possibly reflecting less salivary stimulation or smaller glands, whereas Surdacka^34^ and our study found no flow rate disparity. This inconsistency underscores the need to consider saliva collection methodology and individual variation when comparing results across studies.

Another key reference point is the work of Noorkhakim et al^21^ (2018), who specifically examined salivary calcium and phosphate in Indonesian children with BTS. Their study, involving young children (ages 4–8), found that salivary calcium and inorganic phosphate levels were significantly higher in BTS (+) children than in controls. The authors postulated that this excess of calcium/phosphate could enhance the saliva’s buffering power and help maintain a higher resting pH, thereby creating conditions both for black stain deposition and for caries resistance. Notably, Noorkhakim’s results closely parallel Surdacka’s earlier findings,^34^ reinforcing the concept of a consistent ‘high-mineral, high-pH’ saliva phenotype in BTS-affected children. In contrast, our study did not observe significant Ca or P elevations. This might be due to the older age range of our cohort (mixed–dentition children 7–12 years old) or other population differences; it is conceivable that salivary mineral discrepancies associated with BTS are more pronounced in younger children or diminish as diet and environment diversify with age. Nevertheless, the convergence of evidence from Surdacka and Noorkhakim suggests a real phenomenon: BTS saliva tends to carry higher concentrations of calcium and phosphate than non-BTS saliva in many cases. Our null findings for Ca/P, therefore, should be interpreted with caution and warrant further investigation. It is possible that factors such as widespread fluoride exposure or nutritional status levelled the baseline salivary mineral content between groups, masking differences noted elsewhere.

Intriguingly, our study did identify significantly elevated salivary zinc and manganese in the BTS(+) group; these factors were not highlighted in prior BTS studies. Garan^8^ didn’t measure Zn or Mn, and Surdacka^34^ in fact reported no difference in salivary Zn between the BTS and control groups. The elevated Zn in our BTS children may hold relevance to the low caries incidence, as zinc is known to have antimicrobial properties and can inhibit acidogenic bacteria in plaque.^11^ We speculate that higher salivary Zn could contribute to a less cariogenic biofilm by suppressing *Streptococcus mutans* growth or glycolysis. Manganese, on the other hand, is an essential cofactor for bacterial enzymes; interestingly, some oral bacteria (including certain streptococci) require manganese for virulence, whereas others may be inhibited by excess metal ions.^1^ The role of Mn in BTS is unclear, but one could postulate that elevated salivary Mn might selectively influence the plaque microbiota or interact with the plaque matrix to facilitate the black pigment formation. Nevertheless, it must be emphasised that the literature on Zn and Mn specifically in relation to BTS remains limited. However, the notion that trace elements might modulate BTS has recently been highlighted in a systematic review,^40^ which suggested that salivary copper could play a role in the development of black stains. Our findings open avenues for future research to investigate whether elements such as Zn, Cu, and Mn accumulate in black stain plaques, and how these trace elements might influence bacterial composition and enamel demineralisation rates.

The study by Ortiz-López et al^22^ (2018) provides additional context, especially regarding iron and pH in BTS. Although Ortiz-López’s work focused on adolescents and adults, its insights are relevant. They identified three key factors associated with black stain presence: consuming water with high iron content, consuming water with high alkalinity, and having a naturally high salivary pH. These findings imply that an iron-rich, alkaline oral environment predisposes individuals to developing the black stain. This aligns with the classic understanding that black extrinsic stain is composed of ferric compounds (iron) precipitated under basic conditions. One striking observation was that individuals with BTS actually had lower free iron in their saliva than controls, despite presumably greater exposure to iron via water or diet. They reported that mean salivary iron levels in the BTS group were approximately 12 times lower than in the unstained group. The authors attributed this to the iron being sequestered in the black plaque itself as insoluble ferric sulfide or other complexes, thereby depleting soluble iron in saliva. Our study, conducted in children, did not find a significant difference in salivary iron between BTS(+) and BTS(–) groups. This could be due to generally low iron levels in children’s resting saliva or differences in nutritional iron sources. Nonetheless, Ortiz-López’s ‘iron paradox’ suggests that while environmental iron availability may trigger black stain formation, the resulting plaque acts as a sink for iron. This could have microbiological repercussions; many oral bacteria require trace iron for their metabolism, and a reduction in freely available iron could selectively disadvantage certain cariogenic microbes.^16^ In effect, the black stain itself might contribute to a less caries-conducive milieu by locking away iron that acidogenic bacteria would otherwise utilise. This hypothesis aligns with our finding of low caries and the known microbial profile of black stain.

Although microbiological analyses were not performed in our study, significantly lower caries rates in BTS(+) children indirectly support the consensus that black tooth stain is associated with a distinctive, less cariogenic biofilm community. Prior studies consistently highlight that black stain plaque contains fewer acidogenic bacteria, such as *Streptococcus mutans*, and more non-cariogenic organisms like *Actinomyces* species.^5,30^ Physiologically, the saliva in BTS cases generally exhibits higher pH, increased calcium and phosphate levels, and enhanced buffering capacity, conditions known to support remineralisation and inhibit enamel demineralisation.^8,21,23,34^ Our findings of elevated salivary Zn and Mn in BTS(+) children may further enhance enamel protection, as zinc can inhibit demineralisation and suppress plaque acidity. Additionally, black stain itself, containing iron, calcium, and phosphate minerals, could serve as a localised reservoir for buffering plaque acids, thereby contributing directly to the reduced caries observed. Collectively, these salivary conditions – alkaline pH, mineral richness, and presence of protective trace elements – create a favourable oral environment that inhibits cariogenesis despite the aesthetically undesirable presence of black stain.

This study has several limitations that should be considered when interpreting the results. Firstly, the relatively modest sample size may limit the generalizability of our findings. Another limitation is the absence of data on potential confounding factors such as dietary habits, oral hygiene practices, fluoride exposure, and socioeconomic background, which could influence both saliva composition and caries risk. Finally, microbiological analyses of dental plaque associated with black tooth stain were not performed, which might have provided deeper insights into the mechanisms underlying the observed associations. Future longitudinal studies with larger sample sizes and comprehensive data collection, including microbiological assessment, are recommended to clarify these relationships.

Our findings reinforce the clinical importance of recognising black tooth stain (BTS) as a potential indicator of reduced caries risk in children. Understanding the potential clinical significance of BTS could help dental practitioners better stratify caries risk among paediatric patients. Clinicians might consider incorporating the presence of BTS into individualised caries prevention and monitoring strategies, potentially optimising resource allocation and patient management. Although aesthetically undesirable, BTS presence could reassure clinicians and parents regarding the child’s oral health status. Regular professional prophylaxis may be necessary due to cosmetic concerns, but aggressive preventive interventions might be less warranted for BTS(+) children. Future longitudinal studies are needed to clarify if BTS actively contributes to caries resistance or merely reflects an inherently protective oral environment.

## CONCLUSION

In summary, our study demonstrates that children with BTS exhibit significantly lower caries experience, particularly in primary dentition. While major salivary parameters such as calcium, phosphate, and pH did not significantly differ between groups in our cohort, elevated salivary zinc and manganese levels identified here suggest a novel avenue of investigation into trace elements’ role in black stain formation and caries resistance. Continued research into salivary biochemical profiles and their clinical implications could further clarify the mechanisms underlying BTS, potentially guiding innovative preventive dental strategies.

### Significance of the Study

This investigation represents the first application of the ICDAS II criteria for caries detection in children during the mixed-dentition phase, presenting with black extrinsic tooth stain. Moreover, it is one of the few studies to perform comprehensive salivary biochemistry and trace-ion analysis in paediatric subjects exhibiting this form of extrinsic discolouration.

### Acknowledgements

This study was supported by the Pamukkale University Scientific Research Projects Coordination Unit (Project No: 2021DİŞF002).

## REFERENCES
